# Indian monsoon variability on millennial-orbital timescales

**DOI:** 10.1038/srep24374

**Published:** 2016-04-13

**Authors:** Gayatri Kathayat, Hai Cheng, Ashish Sinha, Christoph Spötl, R. Lawrence Edwards, Haiwei Zhang, Xianglei Li, Liang Yi, Youfeng Ning, Yanjun Cai, Weiguo Lui Lui, Sebastian F. M. Breitenbach

**Affiliations:** 1 Institute of Global Environmental Change, Xi’an Jiaotong University, Xi’an 710049, China; 2 Department of Earth Sciences, University of Minnesota, Minnesota 55455, USA; 3 Department of Earth Sciences, California State University Dominguez Hills, CA 90747, USA; 4 Institut für Geologie, Universität Innsbruck, Innrain 52, A-6020 Innsbruck, Austria; 5 State Key Laboratory of Marine Geology, Tongji University, Shanghai 200092, China; 6 Institute of Earth Environment, Chinese Academy of Sciences, Xi’an 710054, China; 7 Department of Earth Sciences, University of Cambridge, Downing Street, CB2 3EQ Cambridge, UK; 8 institute for Geology, Mineralogy & Geophysics, Ruhr-Universität Bochum, Universitätsstr, 150, 44801 Bochum, Germany

## Abstract

The Indian summer monsoon (ISM) monsoon is critical to billions of people living in the region. Yet, significant debates remain on primary ISM drivers on millennial-orbital timescales. Here, we use speleothem oxygen isotope (δ^18^O) data from Bittoo cave, Northern India to reconstruct ISM variability over the past 280,000 years. We find strong coherence between North Indian and Chinese speleothem δ^18^O records from the East Asian monsoon domain, suggesting that both Asian monsoon subsystems exhibit a coupled response to changes in Northern Hemisphere summer insolation (NHSI) without significant temporal lags, supporting the view that the tropical-subtropical monsoon variability is driven directly by precession-induced changes in NHSI. Comparisons of the North Indian record with both Antarctic ice core and sea-surface temperature records from the southern Indian Ocean over the last glacial period do not suggest a dominant role of Southern Hemisphere climate processes in regulating the ISM variability on millennial-orbital timescales.

The Indian summer monsoon (ISM), as part of the large-scale Asian monsoon (AM) circulation system, transports large amounts of heat and moisture across the equator from the Indian Ocean to South Asia and as far deep as eastern China^1^. To date, significant debates exist with respect to identifying the primary drivers of the ISM variability on millennial^1^,^2^,^3^ to orbital^4^,^5^,^6^ timescales due, in large part, to uncertainties in reconstructing the different physical aspects of monsoon-related variability with various proxies. In the last few decades, results from many climate model simulations indicate that orbital-scale variations in AM strength vary nearly in-phase with changes in precession-dominated NHSI^7^,^8^,^9^,^10^,^11^. These results are supported by speleothem δ^18^O records from southeastern China, which place the East Asian monsoon (EAM) variability nearly in-phase at precession bands with July NHSI when the modern Asian summer monsoon intensity reaches its peak^1^,^12^,^13^ (Fig. 1). Together, these studies support the notion that the global monsoon variability, including the AM, is driven effectively by the land-sea temperature contrast, which in turn, respond sensitively to insolation change. On the other hand, however, numerous proxy records from the Arabian Sea indicate large discrepancies (~8 to 10 ka [thousand years]) between phase estimates of precession-induced changes in NHSI and ISM variability, the latter inferred mainly from reconstructions of wind strength and depth of the oxygen minimum zone^14^,^15^. This lag has been attributed to the cross-equatorial latent heat transport from the southern Indian Ocean, which acts in conjunction with the global ice volume and NHSI, in regulating the ISM variability on orbital timescales^14^,^15^.

To reconcile the contrasting phase estimates, it has been argued that the δ^18^O signatures in the Chinese speleothem records reflect seasonality in the amount and δ^18^O of precipitation, derived from different moisture sources with distinct δ^18^O signatures^4^. Furthermore, by deconvoluting seasonal components of speleothem δ^18^O records, it has been suggested that the summer component of the δ^18^O records (i.e., contributed by rainfall δ^18^O originated from the ISM) lags NHSI by ~8 ka at precession bands and is thus in phase with the Arabian Sea records^4^,^15^. Additionally, a number of studies indicate that upstream changes in δ^18^O of precipitation (δ^18^O_p_) in the ISM domain (rather than local precipitation variability) drive summertime δ^18^O_p_ variations in southeastern China^4^,^16^. Hitherto, it has not been possible to test these hypotheses due to the absence of a summertime δ^18^O_p_ record from the typical ISM domain, which can be independently used to estimate the phasing of ISM δ^18^O_p_ changes relative to NHSI^1^.

Changes in Southern Hemisphere (SH) high-latitude temperature have also been proposed to influence the ISM variability over orbital and millennial timescales^14^. Reconstruction of Pleistocene ISM variability from lake sediments in Heqing basin, southern China suggests that the ISM minimum predated the global ice volume maximum by ~14–35 ka^5^ and its subsequent recovery occurred in the context of SH high-latitude cooling. These observations imply that cooler SH temperatures produce stronger ISM by enhancing the cross-equatorial atmospheric pressure gradient^5^, which appears to be in contrast to the idea that warmer SH temperatures promote the cross-equatorial latent-heat/moisture transport, producing stronger ISM^14^. Additionally, on the basis of comparisons between AM variability mainly from the low-resolution Hulu δ^18^O record^17^ and Antarctic temperature changes during the glacial period^2^,^3^, it has been suggested that SH climate processes dominate the millennial AM variability. However, such comparisons remain tentative because, besides complex precipitation seasonality^4^, the overall δ^18^O signal in Chinese speleothems is relatively small on the millennial timescale and thus possibly compromised by relatively large local signals or noises in their detail structures^1^. In addition, a recent improved high-resolution Hulu record indeed characterized the AM with more abrupt millennial events^18^ than the previous low-resolution data^17^.

In order to address the aforementioned fundamental issues, we developed a long (~280 ka, covering last three glacial-interglacial cycles) speleothem δ^18^O record of the ISM variability^19^ from Bittoo cave in North India (30°47′25″N, 77°46′35″E, ~3000 m.a.s.l.). The cave is located on the fringe of the ISM domain (Supplementary Fig. 1) with ~80% of annual precipitation (~1600 mm) falling during the summer (June to September). Peak summer rainfall in the study area occurs when a strong southeasterly flow develops over the central northern India, serving as a primary conduit for moisture from the Bay of Bengal towards the northwest and northcentral India^20^ (Supplementary Fig. 1). Our analyses of δ^18^O_p_ and low-level monsoon wind trajectory patterns suggest that δ^18^O_p_ variability in the study area results from large-scale upstream changes in monsoon circulation and moisture transport history^19^. These observations agree well with results from model simulations, which show that the δ^18^O_p_ variability in the region is inversely correlated with the ISM intensity on both millennial^16^ and orbital^8^,^9^,^21^ timescales.

The Northern Indian record is a composite of seven stalagmite δ^18^O records based on 167 ^230^Th dates and ~6100 δ^18^O measurements with an average temporal resolution of 50–100 years (Supplementary Figs 2–4, Tables 1 and 2). A strong degree of replication among various speleothem δ^18^O profiles (Supplementary Figs 3 and 4) suggests near-equilibrium growth of stalagmite calcite^1^. The record is characterized by abrupt excursions to extremely low δ^18^O values at rising limbs of NHSI (e.g., at ~109, ~191 and ~243 ka BP [before present, present = 1950 AD]), followed by hiatuses that coincide with intervals of high NHSI during interglacial periods (Fig. 1). In contrast, speleothem growth during the glacial times is continuous and extensive. One plausible explanation to account for initial jumps to extremely low δ^18^O values and subsequent hiatuses during high NHSI periods might involve a marked and abrupt intensification of ISM with depleted δ^18^O signatures. Additionally, stronger rainfall during the interglacial periods in the region may have either lead to undersaturation of Ca^+2^ in dripwater or flooding in Bitto cave, resulting in cessation of speleothem growth. Interestingly, we have also observed a similar pattern of speleothem growth/hiatuses from Hulu cave in China, which share broad geomorphologic similarities with Bittoo Cave (Supplementary Fig. 4).

The Northern Indian δ^18^O record is characterized by a large δ^18^O range (−2 to −12%). Although discontinuous in time, it broadly follows NHSI over the last 280 ka. The δ^18^O record is punctuated by numerous millennial-scale events (namely, the Indian Stadials/Interstadials), which correlate to their counterparts in Greenland^22^ and Chinese speleothem records^13^,^23^ (Fig. 2 and Supplementary Fig. 5). The magnitude of the positive excursions in our record (~5% for Heinrich, Dansgaard/Oeschger (D/O) and Younger Dryas (YD) events) is more than twice larger than those observed in the Chinese δ^18^O records. Nearly all stadials and periods of low NHSI in our record are characterized by a similarly high δ^18^O value (*ca.* −4%), which suggests near-cessation of ^18^O depleted ISM rainfall possibly due to the peripheral location of the study area within the ISM domain (Supplementary Fig. 1). This scenario is consistent with model simulations^16^,^24^ and modern meteorological observations^19^ over the area.

The Northern Indian and Chinese speleothem records^25^ share broad similarities (*r* = ~0.39, p < 0.001) (Supplementary Fig. 6). Both records exhibit the similar structure of the YD, D/O and Heinrich events, and particularly the distinct peak around 18 ka BP, which is neither observed in Greenland nor Antarctic ice cores (Fig. 2). It is also evident that the Marine Isotope Stage (MIS) 5e and well-dated MIS 3 portions in the Northern Indian record show no visible phase difference to NHSI or to its counterparts in EAM records^12^,^13^ (Fig. 2), suggesting nearly in-phase variability at precession bands. Although the Bittoo Cave record encompasses several hiatuses, making a robust statistical analysis difficult, the above observations indeed provide a strong test of the ISM precession variability, as the issue we addressed here is really an 8–10 ka lag versus nearly in-phase (to July NHSI) change on precession scale. In other words, since the MIS 5e and 3 are typical interglacial and glacial periods respectively, the in-phase relation of the ISM with NHSI during these two time periods precludes another relation along with a significant temporal lag, unless the phase relation is completely nonstationary across both interglacial and glacial periods, which however lacks a support of physical mechanism. Our data, together with other fragmentary ISM speleothem records (Fig. 1 and Supplementary Fig. 7) and EAM records^1^,^9^,^12^,^13^,^26^, support the view that speleothem δ^18^O (or δ^18^O_p_) variations are broadly in-phase between the two monsoon subsystems on both orbital and millennial timescales, although seasonal precipitation patterns differ depending on location in this vast monsoon region^1^. Additionally, a recent Chinese speleothem δ^18^O record from Xiaobailong cave from a transitional area between ISM and EAM domains^20^ displays a much larger glacial-interglacial amplitude (e.g., ~4% between MIS 3 and 5)^27^ than both our Northern Indian record and other Chinese speleothem records (~2% or less between MIS 3 and 5). However, the Xiaobailong record also demonstrates generally similar patterns and same timings in comparison with our Northern Indian record (r = 0.57, p < 0.001) (Supplementary Figs 2 and 6) and Chinese EAM records^27^ on both precession and millennial scales.

The covariance between ISM and EAM speleothem δ^18^O records is consistent with model simulations^7^,^8^,^9^,^10^,^11^,^16^,^24^. Additionally, previous studies have demonstrated a strong link between the EAM δ^18^O_p_ and atmospheric δ^18^O (δ^18^O_atm_), possibly through leaf water δ^18^O change^28^. A broad similarity is also observed between the Northern India δ^18^O record and δ^18^O_atm_ records (Fig. 1). If the AM exerts major control on δ^18^O_atm_ variations^28^, one would expect an approximate in-phase, rather than out-of-phase (or anti-phase) relationship between the ISM and EAM at precession bands. In the latter case, the overall AM influence on δ^18^O_atm_ will be largely canceled^1^ and thus, the striking similarity observed among the EAM, ISM and δ^18^O_atm_ records (Fig. 1) will be difficult to reconcile. Our finding thus further supports the hypothesis^7^ that low-latitude monsoon response to insolation shows no significant lag (Supplementary Figs 8–10).

The Northern Indian record provides a critical test on hypotheses concerning the role of cross-equatorial latent heat transport and the pressure gradient in pacing orbital-scale ISM variability. It has been hypothesized that a warmer southern Indian Ocean during *boreal* summer could provide significantly more latent heat to South Asia and thus intensifies the ISM with a temporal pattern that should be significantly out of phase with NHSI on the precessional timescale. This is because a warm southern Indian Ocean presumably lags high NHSI by nearly half a precession cycle (~10 ka)^6^,^14^. On the other hand, the Lake Heqing sediment record from southern China suggests that an overall cooler austral ocean and/or Antarctica strengthen the ISM via enhanced cross-equatorial pressure gradient. The Heqing record, however, lacks data from the last glacial period, which is critical for testing the SH role on the AM. Similar to the EAM speleothem records^12^,^13^, the Northern Indian record shows an ISM intensification after Heinrich event 6 (H6) at ~60 ka BP, which is approximately 40 ka prior to the Last Glacial Maximum (LGM) (Fig. 3), consistent with similar events observed in the Heqing record in previous glaciations^5^. In contrast, the sea-surface temperature records from the southern Indian Ocean^29^ and Antarctic ice cores^30^ indicate that the SH high latitudes underwent slight and gradual warming ~60 ka BP (Fig. 3). Another notable early ISM and EAM rise during the last glacial occurred at ~22 ka BP, ~2.5 ka prior to the LGM and ~3 ka after the Antarctic minimum temperature at ~25 ka BP^30^. However, these two early monsoon enhancements during the last glacial period coincide well with rising NHSI (Fig. 3).

The millennial events in our record have larger amplitudes than their Chinese counterparts, which allow a robust assessment of whether the ISM variability is dominantly controlled by NH or SH high-latitude climate forcing (Fig. 2). In particular, the two events corresponding to Greenland events 15a and 15b around 55 ka BP^22^ manifest clearly in our records (replicated in two different δ^18^O profiles) as well in the EAM^31^ and central European temperature^32^ records (Fig. 2). In contrast, the temperature over Antarctica shows a low-amplitude warming trend from 56 to 54 ka BP. This scenario, particularly the same pattern of Bittoo and the northern Alps records^33^ (Fig. 2 and Supplementary Fig. 7), is consistent with a major NH role in causing these abrupt changes in monsoon systems^34^. Furthermore, Indian stadials (interstadials) in our record are associated with increasing (decreasing) Antarctic temperatures and decreasing (increasing) dust loadings, thus manifesting a typical ‘bipolar seesaw’ pattern^35^. This interhemispheric pattern is incompatible with the hypothesis that temperature changes at high SH latitudes drive variations in the AM and or ISM. It is, however, plausible that monsoon changes in tropical-subtropical regions may themselves be an integral part of the ‘bipolar seesaw’ mechanism; and if so, millennial-scale Antarctic temperature changes could be alternatively viewed to be a result of low latitude monsoon changes, rather than a direct cause of it^1^. Indeed, the Antarctic temperature change is typically small and gradual on millennial-scale, and hitherto needs a physical mechanism or an amplifier in order to explain how it dominantly drives the AM variability with a larger and more abrupt nature (Fig. 2 and Supplementary Fig. 7). The similarity between Antarctic temperature and AM δ^18^O records^3^ is most likely a manifestation of their responses to the same forcing: oceanic reorganization, such as sea surface temperature (SST), triggered by changes in the Atlantic meridional overturning circulation^34^, rather than a direct causal link of one to the other^1^.

The Northern Indian record provides critical information with respect to understanding the ISM dynamics on orbital-millennial timescales. Our data reveal that at precession bands ISM variability is virtually in-phase with July NHSI and the EAM as well. While an intrinsic SH impact on the ISM is plausible via changes in cross-equatorial latent-heat/moisture transport and high-latitude temperatures, our analysis of Northern Indian and EAM, NHSI, Greenland temperature, global ice volume and temperature records from the southern Indian Ocean and Antarctica, suggests that the ISM variability is dominated on orbital to millennial scales by NH climate processes.

## Methods

### ^230^Th dating method

The ^230^Th dating work was performed at two laboratories, the Isotope Laboratory at Xi’an Jiaotong University and the Minnesota Isotope Laboratory, University of Minnesota, using Thermo-Finnigan Neptune/Neptune plus multi-collector inductively coupled plasma mass spectrometers (MC-ICP-MS). The methods are identical in both laboratories. We use standard chemistry procedures to separate uranium and thorium for dating^36^. A triple-spike (^229^Th–^233^U–^236^U) isotope dilution method was employed to correct for instrumental fractionation and determine U/Th isotopic ratios and concentrations. The instrumentation, standardization and half-lives are reported in refs 37 and 38. All U/Th isotopes were measured on a MasCom multiplier behind the retarding potential quadrupole in the peak-jumping mode. We followed similar procedures of characterizing the multiplier as described in ref. 37. Uncertainties in U/Th isotopic data were calculated offline at 2σ level, including corrections for blanks, multiplier dark noise, abundance sensitivity, and contents of the same nuclides in spike solution. Corrected ^230^Th ages assume the initial ^230^Th/^232^Th atomic ratio of 4.4 ± 2.2 × 10^−6^, the values for a material at secular equilibrium with the bulk earth ^232^Th/^238^U value of 3.8. The method and U decay constants are reported in ref. 38. We obtained 167 ^230^Th dates (Supplementary Table 1). Age models for all stalagmites were established using the StalAge program (Supplementary Fig. 2).

### Stable isotope measurements

The oxygen isotopic composition of stalagmite samples was analyzed at four laboratories, Universität Innsbruck, Austria; Nanjing Normal University, China; State Key Laboratory of Loess and Quaternary Geology, Institute of Earth Environment, Chinese Academy of Sciences, and the Isotope Laboratory, Xi’an Jiaotong University, China. A total of ~6100 samples were analyzed using an online carbonate preparation system (Gasbench II) interfaced with an isotope ratio mass spectrometer (Delta^plus^ XL) or a Finnigan MAT-253 mass spectrometer with an on-line carbonate preparation system (Kiel-III or Kiel-IV). Results are reported in per mil (%), relative to the Vienna PeeDee Belemnite (VPDB) standard. Duplicate measurements of NBS19 and TTB1 standards show a long-term reproducibility of ~0.1% (1σ). All δ^18^O data are listed in Supplementary Table S2.

### Statistics Analysis

The correlation coefficients between speleothem δ^18^O records are obtained using the bootstrap resampling method. The composite EAM^25^ and Xiaobailong^27^ records are recalculated based on linear interpolation in order to compare with our North India records on a common timescale. The sample size of each resampling is 500, and the total resampling performed is 2000. The results of statistics analyses are shown in Supplementary Fig. 6.

## Additional Information

**How to cite this article**: Kathayat, G. *et al.* Indian monsoon variability on millennial-orbital timescales. *Sci. Rep.* **6**, 24374; doi: 10.1038/srep24374 (2016).

## Supplementary Material

Supplementary File

Supplementary Data

Supplementary Data

## Figures and Tables

**Figure 1:**
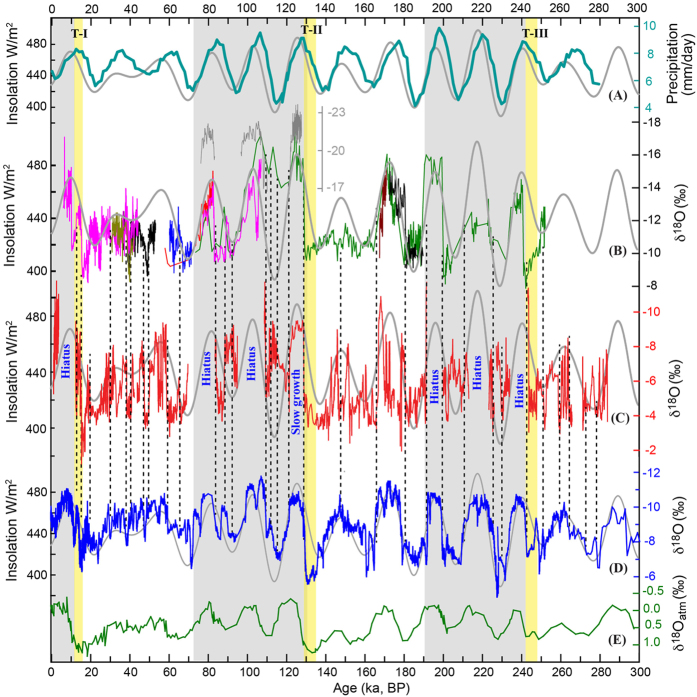
Comparison of ISM and EAM records over the past 280 ka. (**A**) Simulated average summer (June–August) precipitation rate in the ISM region using a fully coupled global ocean–atmosphere model (FOAM)^11^. (**B**) Xiaobailong and Tianmen (grey) records, China^26^,^27^ in the transition area between ISM and EAM domains. The ISM record (this study) and composite EAM record^25^ are shown in red (**C**) and blue (**D**), respectively. (**E**) The atmospheric δ^18^O record from Antarctic ice-core EDC^39^ is plotted for comparison. The grey curves represent July 21 insolation at 65°N^40^. Vertical dashed lines depict correlations of abrupt ISM and EAM shifts. Yellow bars indicate glacial terminations I to III. Grey shadings depict interglacial time periods. Hiatuses occurred mainly during interglacial when NHSI was high. Both ISM and EAM show broadly similar orbital to millennial scale variations, but the ISM record has a larger amplitude. The two monsoon records are similar to the simulation result and follow NHSI broadly on the orbital scale. It is notable that the Xiaobailong record shows much larger glacial-interglacial changes than the Bittoo record.

**Figure 2:**
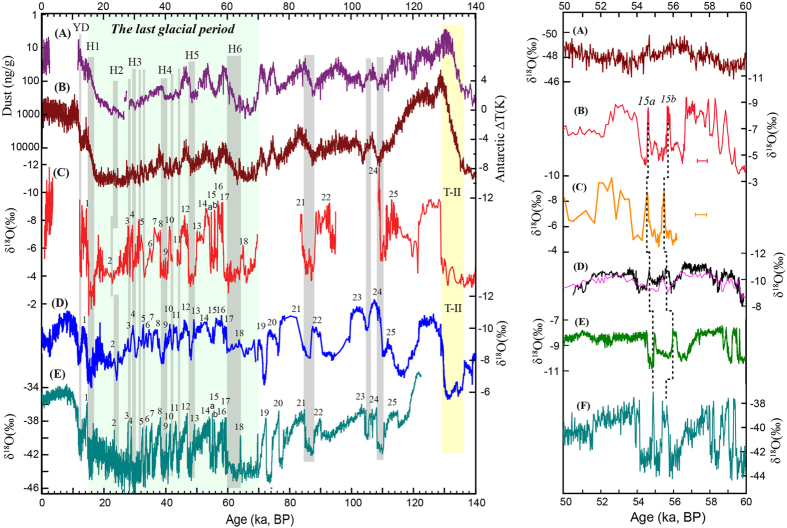
Comparison of climate events in ISM and EAM records over the last 140 ka. Left panel: (**A**) and (**B**) are dust^41^ and temperature^30^ records from Antarctic ice-core EDC, respectively. (**C**) Northern India δ^18^O record. (**D**) EAM δ^18^O record^25^. (**E**) Greenland NGRIP ice-core δ^18^O record^22^. Vertical grey bars indicate weak ISM events and their correlations to weak EAM events, cold events in Greenland, and higher temperature and less dust loading in Antarctica. The yellow bar shows glacial termination T-II. Numbers depict the Indian (**C**), Chinese (**D**) and Greenland (**E**) Interstadials, respectively. These millennial-scale variations are synchronous within age uncertainties. Light-green shading marks the last glacial period. The synchronicities of MIS 3 and MIS 5e between the ISM and EAM demonstrate the in-phase variability of the two monsoon systems on the orbital timescale. Right panel: (**A**) Antarctic ice core EDML δ^18^O record^42^. The δ^18^O scale is reversed as compared with speleothem records. (**B**,**C**) ISM records from Bittoo cave stalagmites BT-2 (red) and BT-1 (orange). Error bars depict typical ^230^Th dating errors (2σ). (**D**) EAM record from Chinese speleothem records (Wulu record in black^31^ and Hulu record in purple^25^). (**E**) The central Europe temperature variation inferred from speleothem records^32^. (**F**) Greenland NGRIP δ^18^O ice-core record^22^. The striking similarity/difference of the ISM variability with/from Greenland/Antarctic records implies a dominant NH rather than SH control on ISM dynamics.

**Figure 3:**
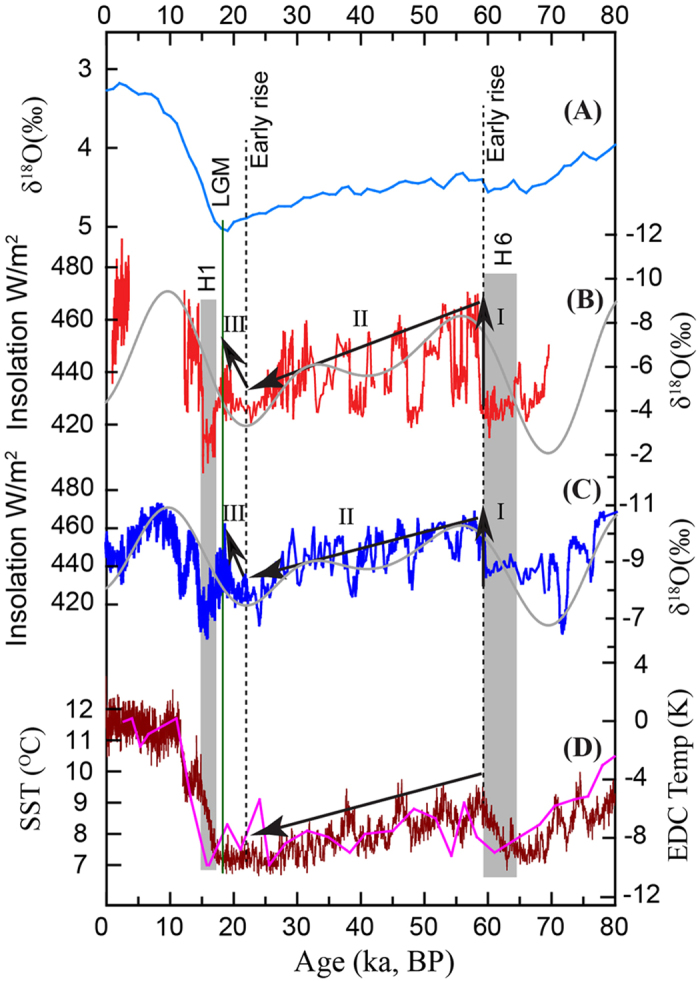
Early ISM increases prior to the LGM during the last glacial period. (**A**) Benthic stack δ^18^O record^43^. (**B**) North India record (red) and July 21 insolation 65°N^40^ (grey curve). (**C**) EAM record^25^ (blue) and July 21 insolation 65°N^40^ (grey curve). (**D**) Antarctic temperature record from ice core EDC^30^ (brown) and southern Indian Ocean SST^29^ (purple). The vertical grey bars depict Heinrich events 1 and 6. Arrow-I marks the abrupt shift of both ISM and EAM at ~60 ka BP when Antarctic temperature reached a maximum about 40 ka prior to the LGM. Arrow-II depicts the ISM and EAM decline concurrently with temperature decreases of both Antarctica and southern Indian Ocean (arrow in **D**) rather than their temperature increases. Arrow-III indicates another early ISM and EAM rise at ~22 ka BP, about 2.5 ka prior to the LGM and about 3 ka after Antarctic temperature reached the minimum at ~25 ka BP. Both early monsoon rises appear to have coincided with NHSI rise rather than with an Antarctic temperature cooling (**D**), thus providing an alternative explanation.
