# Supplementary material for: Indian monsoon variability on millennial-orbital timescales
Source: Sci Rep. 2016 Apr 13;6:24374. doi: 10.1038/srep24374 (PMC4829866; doi:10.1038/srep24374)
Supplement: Supplementary File [file srep24374-s1.pdf]

Indian monsoon variability on millennial-orbital timescales

Gayatri Kathayat[1], Hai Cheng[1,2]*, Ashish Sinha[3], Christoph Spoetl[4], R. Lawrence Edwards[2], Haiwei Zhang[1], Xianglei Li[1], Liang Yi[5], Youfeng Ning[1], Yanjun Cai[6], Weiguo Lui Lui[6], Sebastian F.M. Breitenbach[7]

[1] *Institute of Global Environmental Change, Xi'an Jiaotong University*, Xi’an 710049, China
[2] Department of Earth Sciences, University of Minnesota, Minnesota 55455, USA
[3] *Department of Earth Sciences, California State University Dominguez Hills, CA 90747, USA*
[4] Institut für Geologie, Universität Innsbruck, Innrain 52, A-6020 Innsbruck, Austria
[5] State Key Laboratory of Marine Geology, Tongji University, Shanghai 200092, China
[6] Institute of Earth Environment, Chinese Academy of Sciences, Xi’an 710054, China
[7] Department of Earth Sciences, University of Cambridge, Downing Street, CB2 3EQ Cambridge, UK

Supplementary Information

1. Supplementary Figures

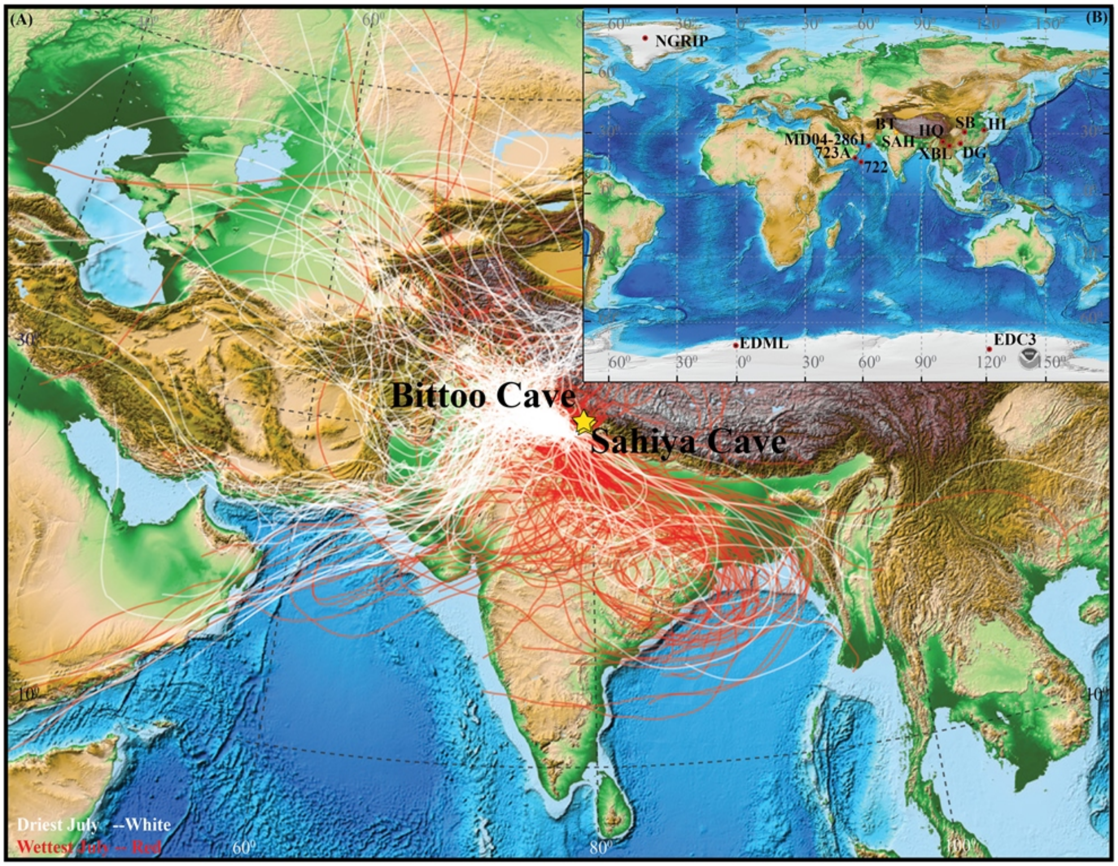

Supplementary Figure 1. Location map. (**A**) Schematic map ETOPO1[1] (Global Relief Model,NOAA http://www.ngdc.noaa.gov/mgg/image/color_etopo1_ice_low.jpg,Computerized digital images and associated databases are available from the National Centers for Environmental Information, National Oceanic and Atmospheric Administration, U.S. Department of Commerce, http://www.ngdc.noaa.gov/.) showing the Bittoo (BT) and Sahiya (SAH) caves (yellow stars) in North India and low-level air parcel trajectories computed from the HYSPLIT model[2]. (**B**) Inset panel shows locations of climate records used

for comparison (red dots): Greenland ice core (NGRIP), Antarctic ice cores (EDML/EDC), Arabian Sea cores (722/MD04-2861/723A), Hulu (HL), Xiaobailong (XBL), Tianmen (TM), Dongge (DG) and Sanbao (SB) East Asian monsoon (EAM) records from Chinese speleothems, and the Heqing (HQ) lake record. The study area receives precipitation mainly from the ISM (~80% of rainfall during JJAS). The red and white lines in (**A**) are multi-year monthly composite of daily backward trajectories originating from the study area during periods of anomalously high (2003, 1985, 1977, 1975, and 1974) and low (2004, 1987, 1970, 1992, and 1986) rainfall, respectively. Anomalously low rainfall in our study area is characterized by near-cessation of ^{18}O-depleted moisture flux from the Bay of Bengal, resulting in the relatively higher flux of ^{18}O-enriched moisture from the local sources and Arabian Sea and dry air intrusions of westerly winds that tend to suppress rainfall over central and northern India through increase of convective stability. On the other hand, anomalously high rainfall in our study area is associated with significantly enhanced transport of ^{18}O-depleted moisture from the Bay of Bengal. The detailed analysis can be found in ref. 3. Modern observations provide a possible analog to the fluctuations recorded in North India $\delta^{18}O$ records, which show similar high $\delta^{18}O$ values when the ISM presumably failed during the ISM stadials or low Northern Hemisphere summer insolation periods, likely indicating that during these time periods the moisture might be mainly derived from the Westerlies. This interpretation is consistent with recent model simulations[4-6].

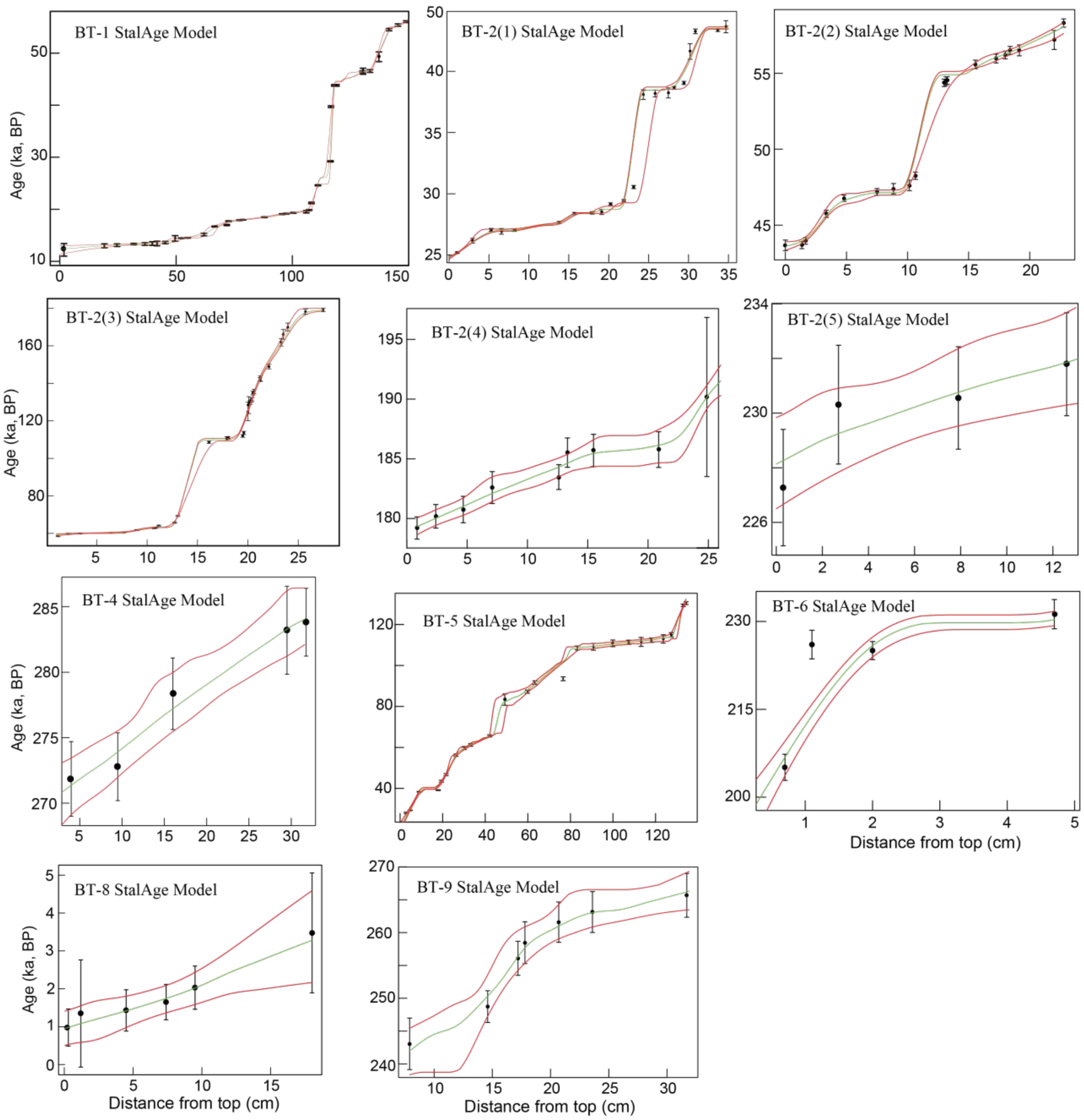

Supplementary Figure 2. Age models of stalagmites from Bittoo cave (BT-1, BT-2, BT-4, BT-5, BT-6, BT-8 and BT-9). The age models are established by using 167 ^{230}Th dates (Supplementary Table S1) and StalAge[7]. The vertical error bars depict ^{230}Th dating errors (2σ). Sample BT-2 is broken into five sections, BT-2(1) to BT-2(5), and the depth of each section starts with 0. There are two hiatuses in sample BT-1 at 46.8 and 116.4 cm depth, respectively. Two hiatuses are present in sample BT-2(1) at 23.95 and 30.15 cm depth, respectively. BT-2(3) grew extremely slow around 13.20 cm (~0.0001 mm/year), equivalent to a hiatus, and another hiatus in the sample occurred at 23.92 cm. BT-5 has two hiatuses at 42.09 and 76.70 mm, respectively. BT-6 has a hiatus at 1.21 cm.

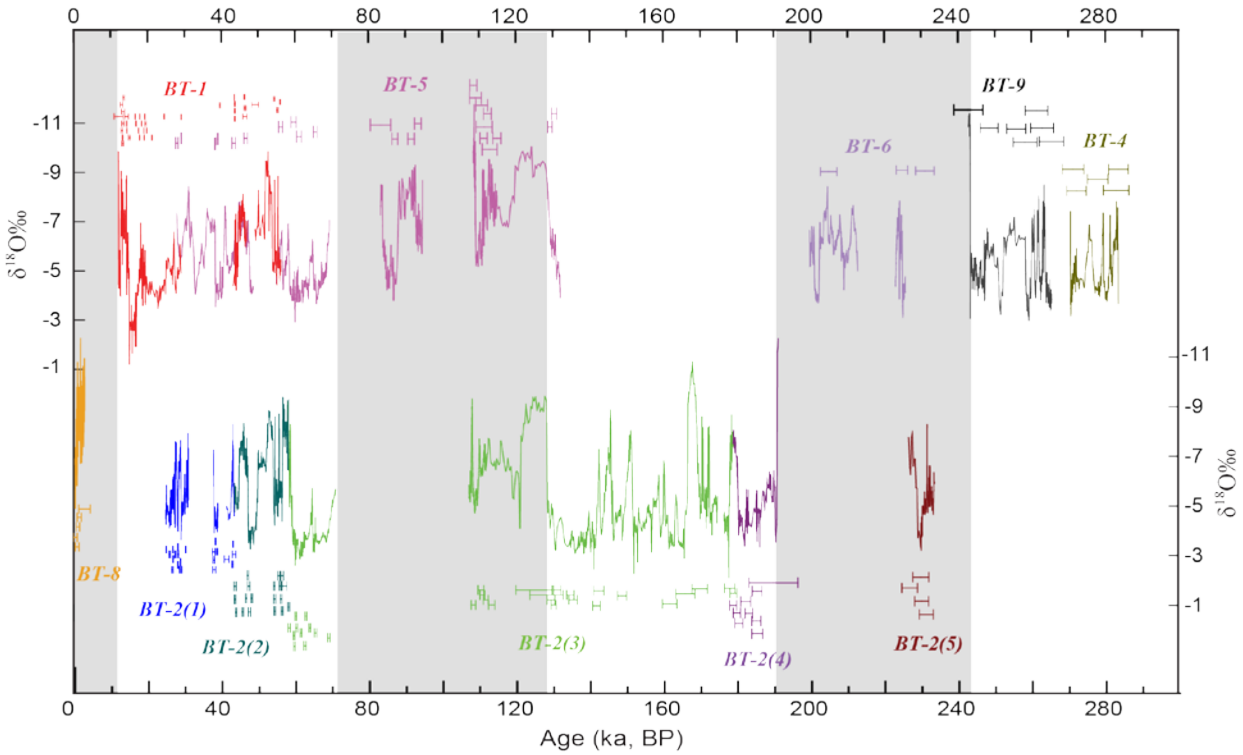

Supplementary Figure 3. δ^{18}O records of stalagmites from Bittoo cave (*BT-1, BT-2, BT-4 to BT-6, BT-8 and BT-9*). Error bars indicate ^{230}Th ages and errors (2σ). The ^{230}Th dating method is described in *ref. 8*. The grey shadings depict interglacial periods. The high degree of coherence among contemporary δ^{18}O profiles (*BT-1, BT-2 and BT-5*) demonstrates that kinetic isotope effects are insignificant[9].

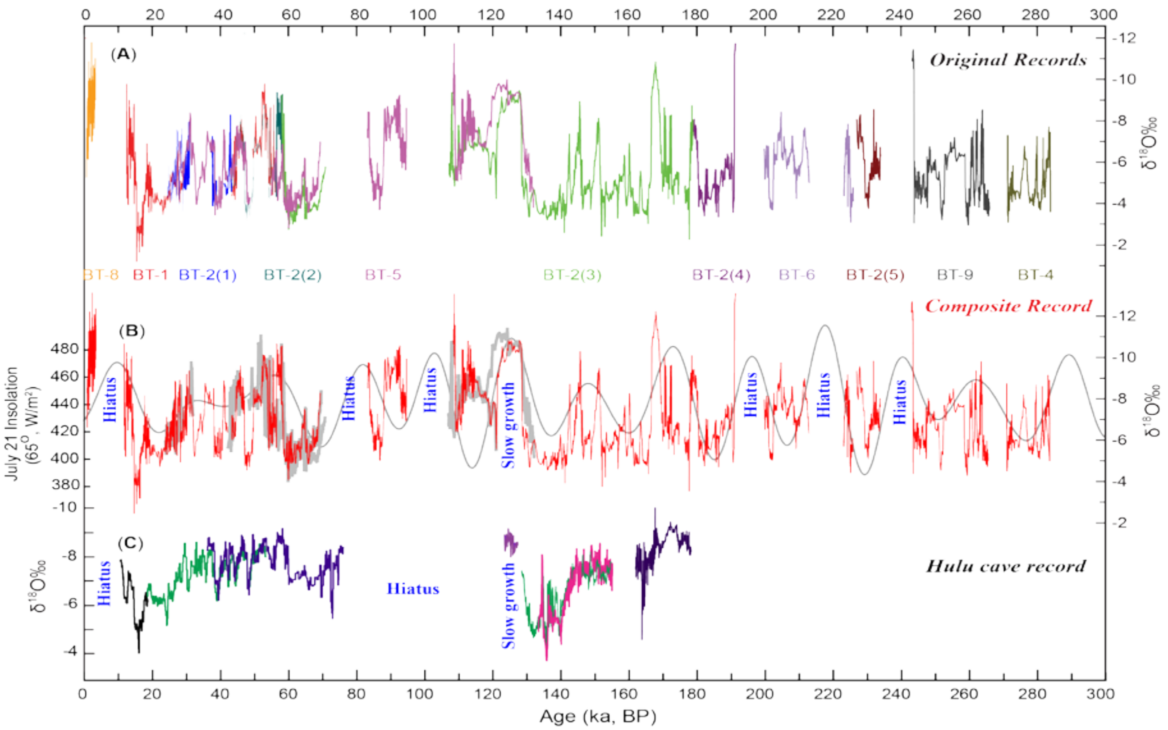

Supplementary Figure 4. Composite $\delta^{18}O$ record over the past 280 ka from Bittoo cave. The composite Bittoo record is constructed by stacking Bittoo cave $\delta^{18}O$ profiles. Two criteria (higher resolution and better dating control) are employed for the selection of records over contemporary growth periods. (**A**) Original $\delta^{18}O$ profiles of seven stalagmites. (**B**) The composite $\delta^{18}O$ record from the seven stalagmites is shown in red. Data in grey are not used in the composite record due to relatively low resolution and/or large age uncertainty (Supplementary Table S1 and S2). The Bittoo record displays very large amplitude fluctuations on both orbital (~10‰) and millennial (~5‰) timescales, which is difficult to be explained by temperature variations. The record broadly follows NHSI and is punctuated by many millennial-scale events. This pattern is similar to the East Asian monsoon record reconstructed from Chinese speleothems[10,11]. (**C**) Hulu cave records, China[10-12]. The Bittoo record is characterized by abrupt $\delta^{18}O$ excursions to extremely low values at rising limbs of the NHSI (e.g. at ~109, ~191 and ~243 ka BP), which are followed by hiatuses that coincide with intervals of high NHSI during interglacial periods. We previously observed the same phenomenon from well-known Hulu cave, China. Hulu cave also lacks speleothem growths during the Holocene and MIS 5, and the only MIS 5 sample dated within the MIS 5e time range also grew extremely slowly[12]. In contrast, the growth during the glacial times in both Bittoo and Hulu caves is continuous and extensive. Given the fact that both Bittoo and Hulu caves are close and small chambers (~20x30 meters), one plausible explanation of the hiatuses occurred during interglacial periods might involve rainfall-induced flooding during stronger monsoon periods inferred from abrupt $\delta^{18}O$ jumps to extremely low values. Consistent with this explanation is the scenario that the $\delta^{18}O$ values exhibit a sharp increase (e.g. at ~65, ~188 and ~234 ka BP) when speleothem growth resumed. This interpretation is consistent with the observation that floods occurred in Bittoo cave after strong rainfall if considering the fact that the modern ISM is presumably weaker than the higher NHSI periods when the major Hiatuses occurred in Bittoo records.

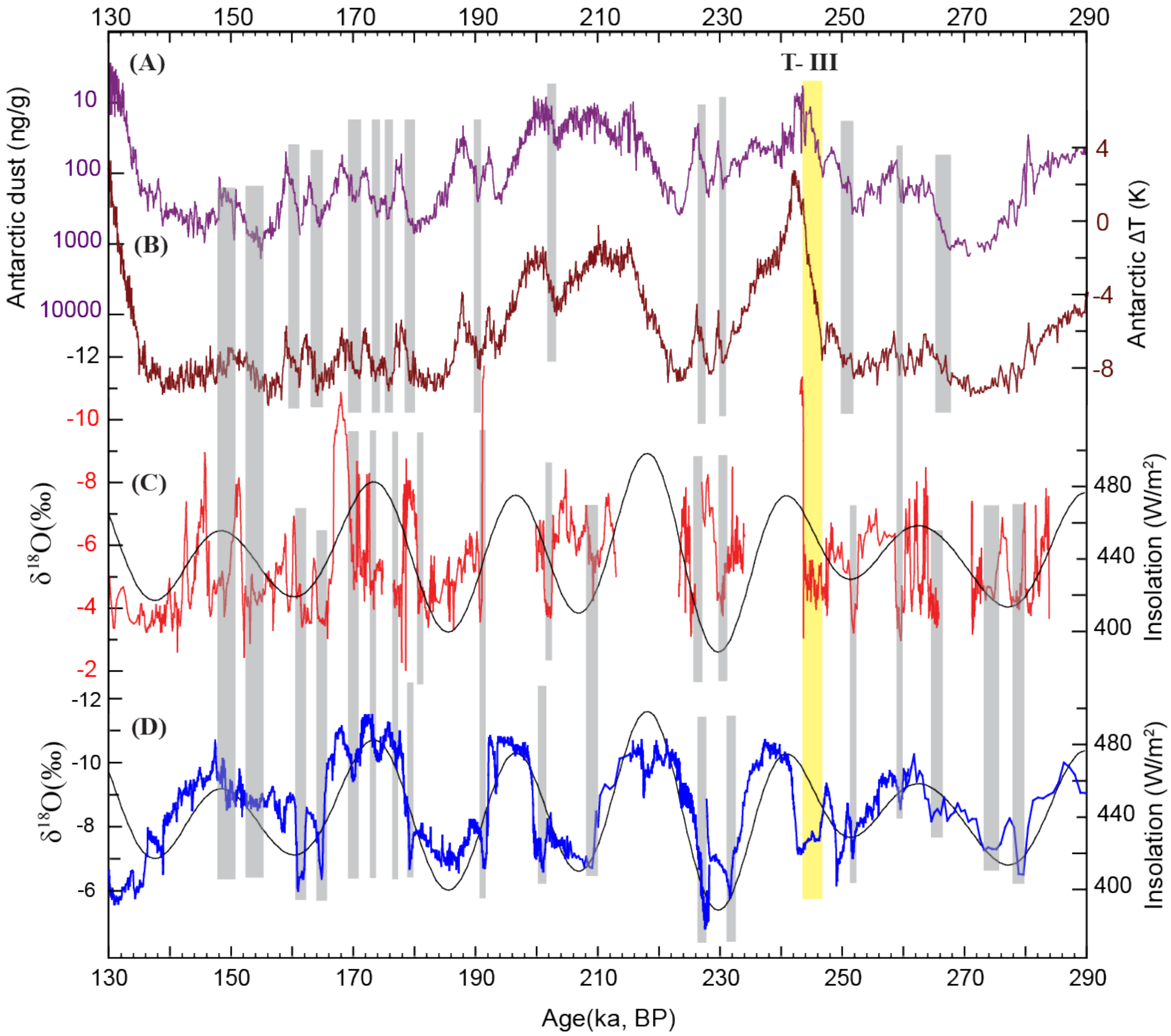

Supplementary Figure 5. Millennial-scale ISM events between 280 and 130 ka BP and their correlations with Antarctic dust and temperature and the EAM records. (**A**) Dust records from Antarctic ice core EDC[13]. (**B**) EDC temperature record[14]. (**C**) Composite Bittoo $\delta^{18}O$ record (red) and July 21 insolation at 65°N[15] (Black). (**D**) EAM speleothem $\delta^{18}O$ record[16]. The yellow bar shows Termination T-III in the ISM record. The grey bars depict millennial-scale events observed in the ISM, EAM and Antarctic dust and temperature records.

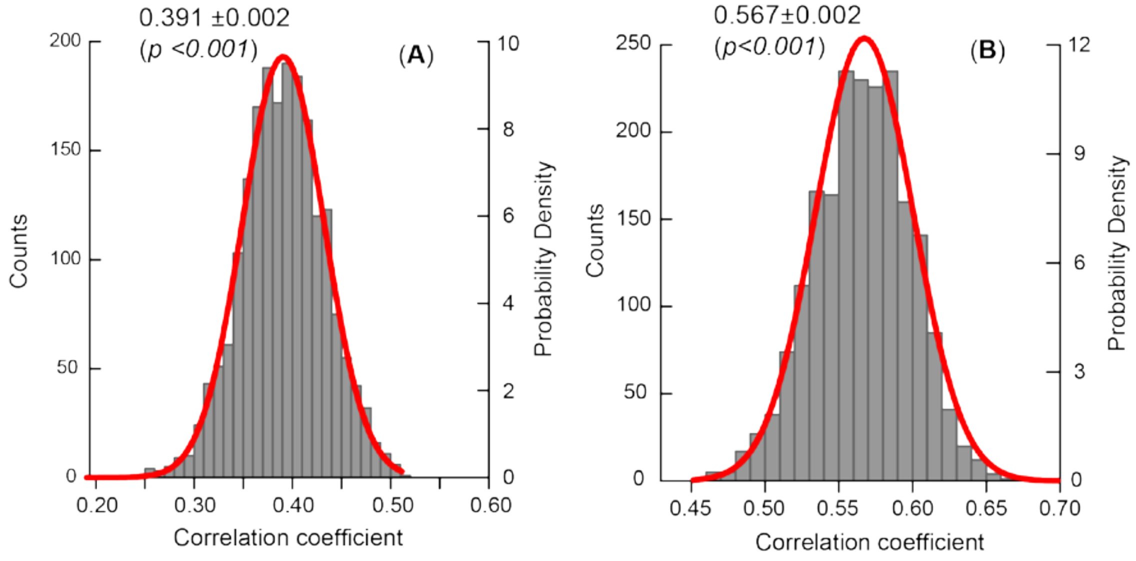

Supplementary Figure 6. Histograms of correlation coefficients between speleothem $\delta^{18}O$ records. The correlation coefficients are calculated by linear interpolation of the speleothem $\delta^{18}O$ data onto a common timescale. The correlation coefficients are obtained by using bootstrap resampling (N = 2000; sample size = 500) between the Indian Monsoon (this study) and East Asian monsoon[17] composite speleothem $\delta^{18}O$ records (**A**) and between the Bitto Cave (this study) and Xiaobailong cave[18] $\delta^{18}O$ records (**B**). The Gaussian distribution (red lines) is used to fit expected probability density curves. The correlation coefficients and their standard errors are labeled at the top. The time period of speleothem records used to analyze is limited over the last 200 ka. This is because uncertainties of absolute ages are significantly larger for these speleothem records older than 200 ka BP (~1.5 to 4 ka).

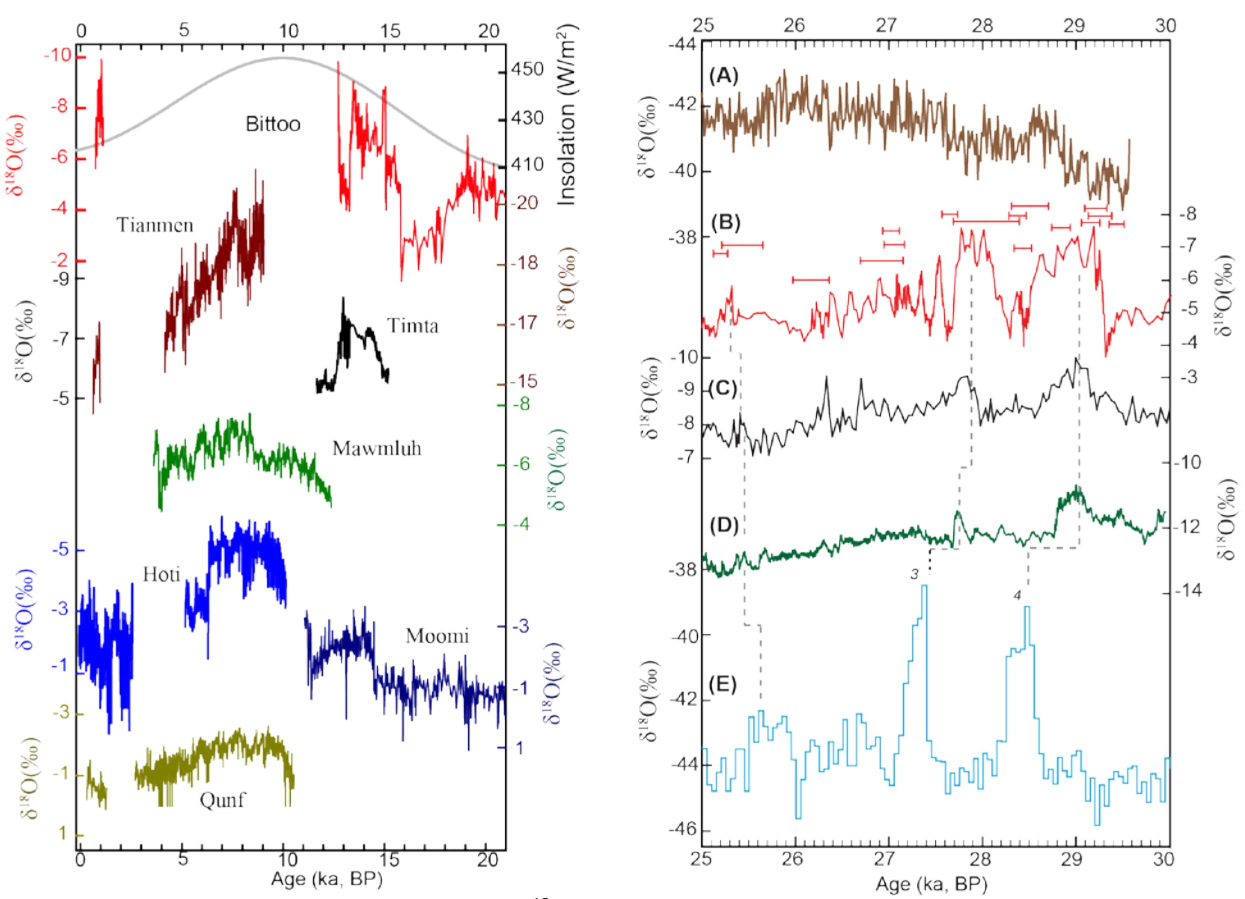

Supplementary Figure 7. Speleothem δ^{18}O records in the wide ISM domain over the past 21 ka (left panel) and a comparison of the Bittoo record with Chinese and European speleothem records, as well as ice core records between 30 and 25 ka BP (right panel). Left panel: The overall patterns of speleothem records (cave names are indicated in the plot) in the ISM region are broadly similar and comparable to that of the East Asian monsoon documented in Chinese speleothem δ^{18}O records (modified from *refs. 5 and 17*). July 21 insolation at 65°N[15] (grey) is plotted at the top for comparison. Right panel: (**A**) Antarctic ice core (WDC) δ^{18}O record[19]. The δ^{18}O scale is reversed as compared with speleothem records. (**B**) The ISM record from Bittoo cave. Error bars depict typical ^{230}Th dating errors (2σ). (**C**) The EAM record from Chinese speleothem record[16]. (**D**) Alpine temperature variation inferred from speleothem records[20]. (**E**) Greenland ice core (NGRIP) δ^{18}O record[21]. The vertical dashed lines indicate the correlation of the major events among different records. It appears to lack an amplifier or mechanism if one links the large monsoon variability causally to the Antarctic temperature changes with a small and gradual nature.

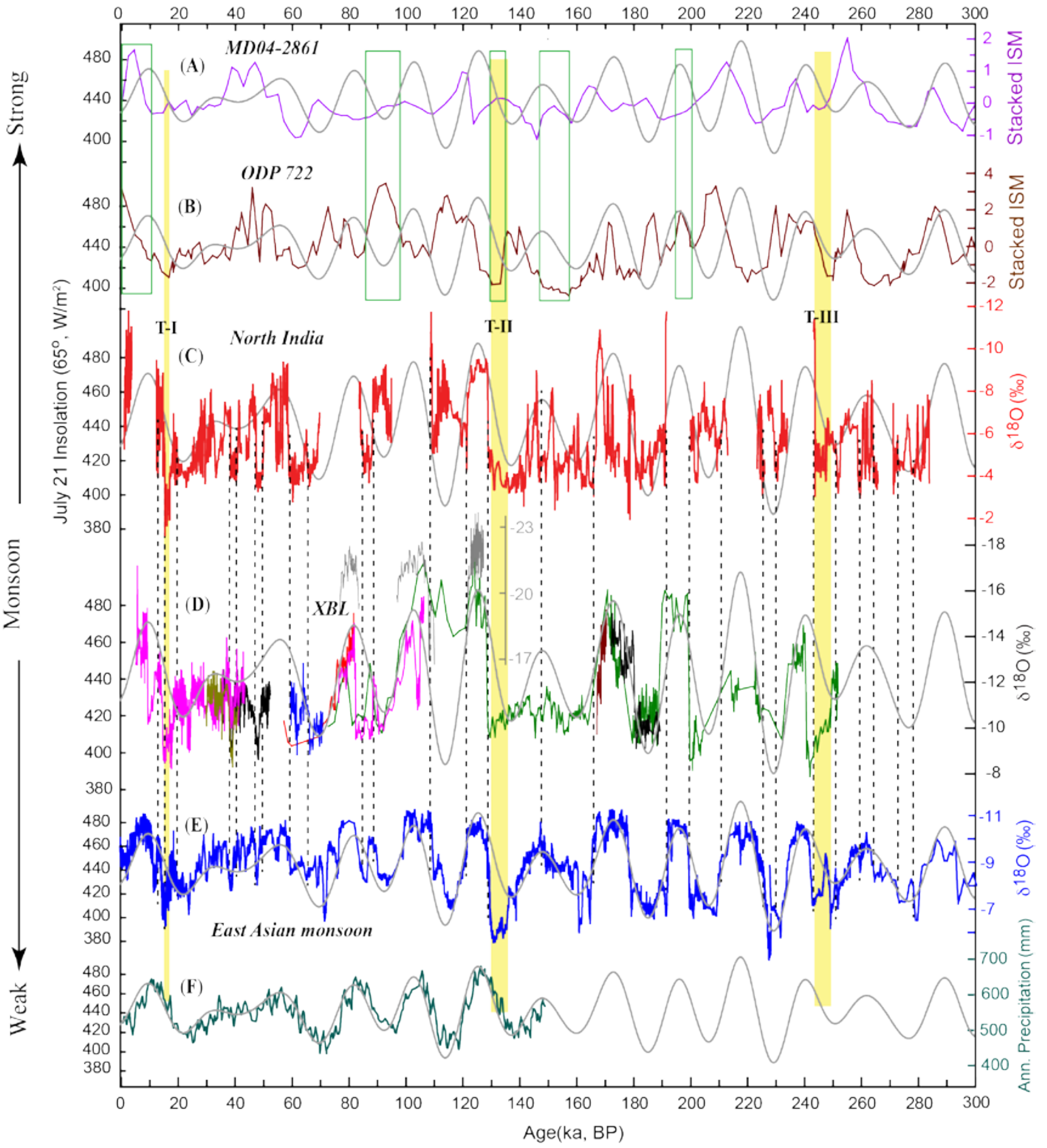

Supplementary Figure 8. Comparison between the Asian monsoon records from marine sediments, speleothems and model simulation. (**A**) and (**B**) are the stacked ISM records from Arabian Sea cores ODP722[22] and MD04-2861[23], respectively. (**C**) Composite Bittoo $\delta^{18}O$ record. (**D**) Xiaobailong[18] (XBL) and Tianmen[24] (grey) records from China. (**E**) EAM $\delta^{18}O$ record[16]. (**F**) Community Climate System Model version 3 (CCSM3) simulated annual precipitation in East Asia[25]. July 21 insolation at 65°N[15] (grey) is plotted for comparison. Dashed lines show common shifts found in both ISM and EAM. Green boxes mark significant discrepancies between the two stacked ISM records from the Arabian Sea. While the ISM and EAM are generally consistent on precessional-millennial timescales in speleothem records and model simulations, the ISM records from Arabian Sea sediments show considerable discrepancies. In other words, the relationship between the marine ISM records and NHSI could be different in different records depending on the locations and/or proxies, as for example, during the Holocene (see Supplementary Figure 9).

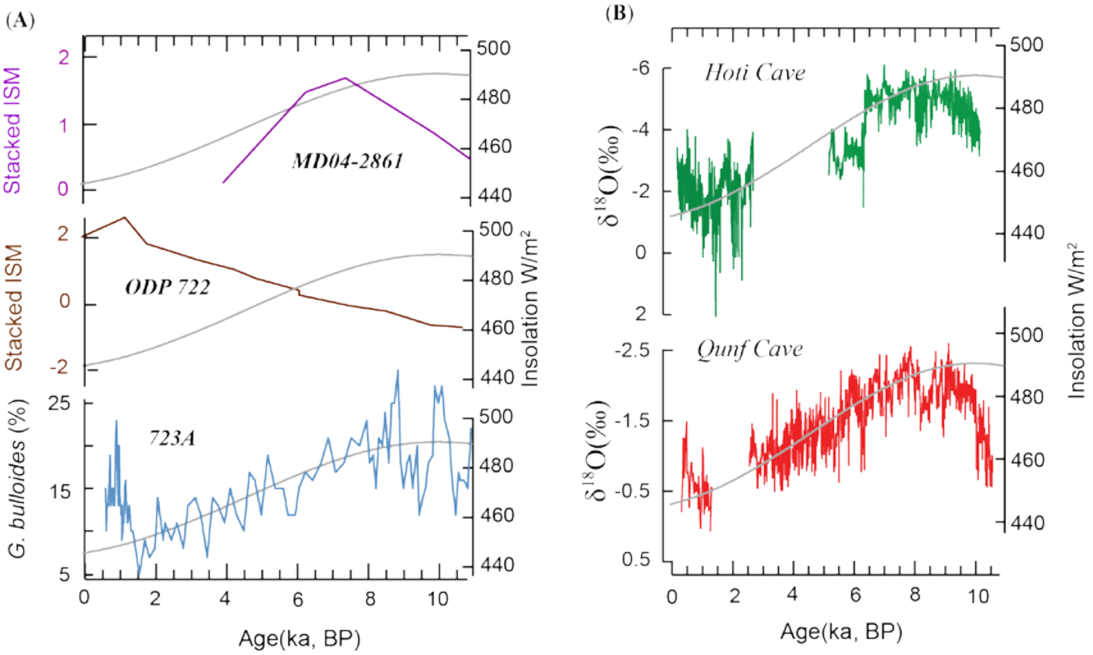

Supplementary Figure 9. Comparison of Holocene Asian monsoon records from marine sediments and speleothems. (**A)** Holocene portions of the stacked ISM records from Arabian Sea cores ODP722[22] (upper) and MD04-2861[23] (middle), respectively. *G. bulloides* (%) from the Arabian Sea core 723A[26] is plotted at the bottom. (**B**) Holocene ISM $\delta^{18}O$ records from Oman[27] (Hoti cave in green and Qunf cave in red). July 21 insolation at 65°N[15] (grey) is plotted for comparison. It is evident that ISM and EAM variations are generally consistent with speleothem records, and in contrast, the three ISM records from Arabian Sea sediments show considerable discrepancies with one of them (core 723A[26]) virtually similar to speleothem records.

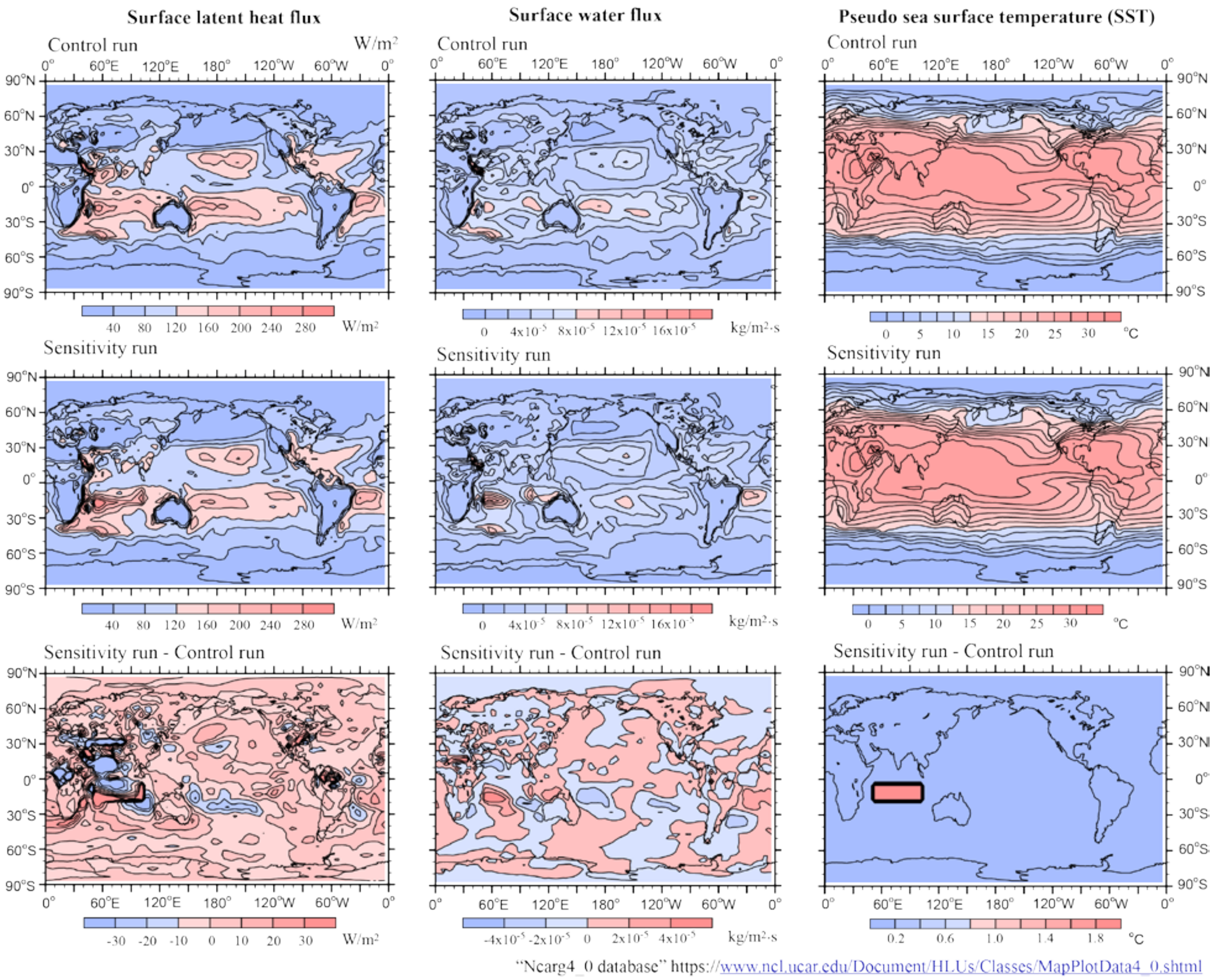

Supplementary Figure 10. Latent heat simulation results from the Community Atmosphere Model (CAM3). The Community Atmosphere Model (CAM3) is analyzed using NCL (Interpreted language designed specifically for scientific data analysis and visualization, "Ncarg4_0 database" https://www.ncl.ucar.edu/Document/HLUs/Classes/MapPlotData4_0.shtml). The visualizations are also created using NCL Software[28]. First to the third column are July latent heat flux, surface water flux and pseudo sea surface temperature (SST) results. First to the third row are results of control run, sensitivity run and sensitivity – control run, respectively. In the sensitivity run, the SST in the subtropical region of the Southern Indian Ocean (50°E-110°E, 5°S-15°S) is increased by 2°C. Both control and sensitivity runs run for a ten-year period and the results of the later five-year average are used for comparison. Our results show that the increase of SST in the tropical Southern Indian Ocean by 2°C will enhance the evaporation in this region and, however, has an insignificant impact on both the Indian monsoon and latent heat transition to the Asian continent.

References

1. Amante, C. and B.W. Eakins, ETOPO1 1 Arc-Minute Global Relief Model: Procedures, Data Sources and Analysis. NOAA Technical Memorandum NESDIS NGDC-24. National Geophysical Data Center, NOAA. doi:10.7289/V5C8276M [23.13.2013] (2009).
2. Draxler, R. R. & Rolph, G. D. HYSPLIT (Hybrid Single-Particle Lagrangian Integrated Trajectory) Model access via NOAA ARLREADY Website http://www.arl.noaa.gov/ready/hysplit4.html NOAA Air Resources Laboratory (2003).
3. Sinha, A. *et al*. Trends, Oscillations and Anomalies in the Indian Summer Monsoon Rainfall over the Last Two Millennia. *Nature Communications*, 6:6309 | DOI: 10.1038/ncomms7309 (2015).
4. Pausata, F. S., Battisti, D. S., Nisancioglu, K. H. & Bitz, C. M. Chinese stalagmite $\delta^{18}O$ controlled by changes in the Indian monsoon during a simulated Heinrich event. *Nature Geoscience* **4**, 474–480 (2011).
5. Liu, Z. *et al.* Chinese cave records and the East Asia summer monsoon. *Quat. Sci. Rev.* **83**, 115–128 (2014).
6. Chiang, J. C. H. *et al*. Role of Seasonal Transitions and Westerly Jets in East Asian Paleoclimate. *Quat. Sci. Rev*. **108**, 111–129 (2015).
7. Scholz, D. & Hoffmann, D. L. StalAge–an algorithm designed for construction of speleothem age models. *Quat. Geochron.* **6**, 369–382 (2011).
8. Cheng, H. *et al.* Improvements in ^{230}Th dating, ^{230}Th and ^{234}U half-life values, and U–Th isotopic measurements by multi-collector inductively coupled plasma mass spectrometry. *Earth Planet. Sci. Lett.* **371**, 82–91 (2013).
9. Hendy, C. H. The isotopic geochemistry of speleothems—I. The calculation of the effects of different modes of formation on the isotopic composition of speleothems and their applicability as palaeoclimatic indicators. *Geochim. Cosmochim. Acta* **35**, 801–824 (1971).
10. Wang, Y. *et al.* Millennial-and orbital-scale changes in the East Asian monsoon over the past 224,000 years. *Nature* **451**, 1090–1093 (2008).
11. Cheng, H. *et al.* Ice age terminations. *Science* **326**, 248–252 (2009).
12. Cheng, H. *et al.* A penultimate glacial monsoon record from Hulu Cave and two-phase glacial terminations. *Geology* **34**, 217–220 (2006).
13. Lambert, F. *et al.* Dust-climate couplings over the past 800,000 years from the EPICA Dome C ice core. *Nature* **452**, 616–619 (2008).
14. Jouzel, J. *et al.* Orbital and millennial Antarctic climate variability over the past 800,000 years. *Science* **317**, 793–796 (2007).
15. Berger, A. Long-term variations of caloric insolation resulting from the Earth's orbital elements. *Quat. Res.* **9**, 139–167 (1978).
16. Cheng, H. *et al*. The climatic cyclicity in semiarid-arid central Asia over the past 500,000 years. *Geophy. Res. Lett*. **39**, L01705, doi:10.1029/2011GL050202 (2012).

17. Cheng, H., Sinha, A., Wang, X., Cruz, F. W. & Edwards, R. L. The Global Paleomonsoon as seen through speleothem records from Asia and the Americas. *Clim. Dyn.* **39**, 1045–1062 (2012).
18. Cai, Y.J. *et al*. Variability of stalagmite-inferred Indian monsoon precipitation over the past 252,000 y. *Proc. Nat. Acad. Sci. U.S*. **112**, 2954–2959 (2015).
19. Members, W. D. P. Onset of deglacial warming in West Antarctica driven by local orbital forcing. *Nature* **500**, 440-444 (2013).
20. Luetscher, M. *et al.* North Atlantic storm track changes during the Last Glacial Maximum recorded by Alpine speleothems. *Nature communication, 6:6344* | DOI: 10.1038/ncomms7344 (2015).
21. Wolff, E. W., Chappellaz, J., Blunier, T., Rasmussen, S. O., and Svensson, A. Millennial-scale variability during the last glacial: The ice core record. *Quat. Sci. Rev*. **29**, 2828–2838 (2010).
22. Clemens, S. C. & Prell, W. L. A 350,000 year summer-monsoon multi-proxy stack from the Owen Ridge, Northern Arabian Sea. *Marine Geol.* **201**, 35–51 (2003).
23. Caley, T. *et al.* Orbital timing of the Indian, East Asian and African boreal monsoons and the concept of a 'global monsoon'. *Quaternary Science Reviews* **30**, 3705–3715 (2011).
24. Cai, Y. *et al.* Large variations of oxygen isotopes in precipitation over south-central Tibet during Marine Isotope Stage 5. *Geology* **38**, 243–246 (2010).
25. Li, X., Liu, X., Qiu, L., An, Z., Yin, Z. Y. Transient simulation of orbital-scale precipitation variation in monsoonal East Asia and arid central Asia during the last 150 ka, *J. Geophys. Res. Atmos*. **118**, 7481–7488 (3013).
26. Gupta, A. K., Anderson, D. M. Overpeck, J. T. Abrupt changes in the Asian southwest monsoon during the Holocene and their links to the North Atlantic Ocean. *Nature* **421**, 354–357 (2003).
27. Fleitmann, D. *et al.* Holocene ITCZ and Indian monsoon dynamics recorded in stalagmites from Oman and Yemen (Socotra). *Quaternary Science Reviews* **26**, 170–188 (2007).
28. The NCAR Command Language (Version 6.3.0) [Software].(2015). Boulder, Colorado:UCAR/NCAR/CISL/TDD. http://dx.doi.org/10.5065/D6WD3XH5

Extended Data Table 1. ^{230}Th dating results.

Extended Data Table 2. Stable isotope data of seven stalagmites from Bittoo cave, North India.
